# Supplementary material for: Increase of 4-Hydroxybenzoic, a Bioactive Phenolic Compound, after an Organic Intervention Diet
Source: Antioxidants (Basel). 2019 Aug 24;8(9):340. doi: 10.3390/antiox8090340 (PMC6769758; doi:10.3390/antiox8090340)
Supplement: Supplementary file 1 [file antioxidants-08-00340-s001.pdf]

Table S1. Baseline concentrations of inorganic elements in plasma (n = 38).

**Elements**	
Na (mg/kg)	2990 ± 15
K (mg/kg)	825 ± 9
Ca (mg/kg)	88 ± 0
Mg (mg/kg)	17 ± 0
P (mg/kg)	104 ± 2
Fe (μg/kg)	1304 ± 69
Zn (μg/kg)	756 ± 24
Cu (μg/kg)	837 ± 45
As (μg/kg)	2.92 ± 0.68
Pb (μg/kg)	BLD
Cd (μg/kg)	BLD
U (μg/kg)	BLD

Date are mean ± SEM; BLD: below limit of detection.

Table S2. Baseline concentrations of urinary excretion of phenolic acids (n = 38).

**Compounds**	
Phenylacetic acids	
3,4-DHPAA (nmol)	68 ± 20
3-HPAA (nmol)	2017 ± 871
Homovanillic (nmol)	157 ± 38
Phenylpropionic acids	
3,4-HPPA (nmol)	33 ± 20
DHCA (nmol)	2.1 ± 0.5
Hydroxybenzoic and derivatives	
4-HBA (nmol)	196 ± 81
4-HH (nmol)	389 ± 125
Hippuric (nmol)	1462 ± 156
Hydroxycinnamic and derivatives	
CA (nmol)	9 ± 1
m-Cou (nmol)	0.6 ± 0.2
p-Cou (nmol)	0.6 ± 0.3

GA (nmol)	1.17 ± 0.87

Data are mean ± SEM. 3,4-DHPAA: 3,4-dihydroxyphenylacetic acid, 3-HPAA: 3-hydroxyphenylacetic acid, 3,4-HPPA: 3-(4-hydroxyphenyl) propionic acid, DHCA: dihydrocaffeic acid, 4-HBA: 4-hydroxybenzoic acid, 4-HH: 4-hydroxyhippuric, CA: caffeic acid, *m*-Cou: *m*-coumaric acid, *p*-Cou: *p*-coumaric acid, GA: gallic acid.

Table S3. Baseline concentrations of carotenes in plasma (n = 38).

**Compounds**	
α-carotene (nmol/mL)	0.35 ± 0.08
β-carotene (nmol/mL)	0.91 ± 0.29
E-Lycopene (nmol/mL)	1.19 ± 0.14
5-*Z*-Lycopene (nmol/mL)	0.26 ± 0.03

Data are mean ± SEM.
